# The rat suprachiasmatic nucleus: the master clock ticks at 30 Hz

**DOI:** 10.1113/JP272331

**Published:** 2016-05-29

**Authors:** Takahiro Tsuji, Chiharu Tsuji, Mike Ludwig, Gareth Leng

**Affiliations:** ^1^ Centre for Integrative PhysiologyUniversity of EdinburghEdinburghUK

## Abstract

**Key points:**

- Light‐responsive neurones in the rat suprachiasmatic nucleus discharge with a harmonic distribution of interspike intervals, whereas unresponsive neurones seldom do.
- This harmonic patterning has a fundamental frequency of close to 30 Hz, and is the same in light‐on cells as in light‐off cells, and is unaffected by exposure to light.
- Light‐on cells are more active than light‐off cells in both subjective day and subjective night, and both light‐on cells and light‐off cells respond more strongly to changes in light intensity during the subjective night than during the subjective day.
- Paired recordings indicate that the discharge of adjacent light‐responsive cells is very tightly synchronized.
- The gap junction inhibitor carbenoxolone increases the spontaneous activity of suprachiasmatic nucleus neurones but does not block the harmonic discharge patterning.

**Abstract:**

The suprachiasmatic nucleus (SCN) of the hypothalamus has an essential role in orchestrating circadian rhythms of behaviour and physiology. In the present study, we recorded from single SCN neurons in urethane‐anaesthetized rats, categorized them by the statistical features of their electrical activity and by their responses to light, and examined how activity in the light phase differs from activity in the dark phase. We classified cells as light‐on cells or light‐off cells according to how their firing rate changed in acute response to light, or as non‐responsive cells. In both sets of light‐responsive neurons, responses to light were stronger at subjective night than in subjective day. Neuronal firing patterns were analysed by constructing hazard functions from interspike interval data. For most light‐responsive cells, the hazard functions showed a multimodal distribution, with a harmonic sequence of modes, indicating that spike activity was driven by an oscillatory input with a fundamental frequency of close to 30 Hz; this harmonic pattern was rarely seen in non‐responsive SCN cells. The frequency of the rhythm was the same in light‐on cells as in light‐off cells, was the same in subjective day as at subjective night, and was unaffected by exposure to light. Paired recordings indicated that the discharge of adjacent light‐responsive neurons was very tightly synchronized, consistent with electrical coupling.

## Key points

- Light‐responsive neurones in the rat suprachiasmatic nucleus discharge with a harmonic distribution of interspike intervals, whereas unresponsive neurones seldom do.
- This harmonic patterning has a fundamental frequency of close to 30 Hz, and is the same in light‐on cells as in light‐off cells, and is unaffected by exposure to light.
- Light‐on cells are more active than light‐off cells in both subjective day and subjective night, and both light‐on cells and light‐off cells respond more strongly to changes in light intensity during the subjective night than during the subjective day.
- Paired recordings indicate that the discharge of adjacent light‐responsive cells is very tightly synchronized.
- The gap junction inhibitor carbenoxolone increases the spontaneous activity of suprachiasmatic nucleus neurones but does not block the harmonic discharge patterning.

**Abbreviations**
ISI: interspike interval
SCN: suprachiasmatic nucleus
ZT: zeitgeber time

## Introduction

In mammals, circadian rhythms are controlled by the suprachiasmatic nuclei (SCN) of the hypothalamus, the ‘master clock’ of the body (Rusak & Zucker, 1979). Lesions to the SCN eliminate circadian rhythms in behaviour, and these rhythms can be restored by implantation of fetal SCN tissue; thus, SCN neurones display an intrinsic circadian rhythmicity (Takahashi *et al*. 2008). In the intact animal, these rhythms are synchronized to the environmental light–dark cycle by information on the intensity and spectral composition of ambient light (Walmsley *et al*. 2015) that is conveyed directly to the SCN by glutamatergic afferents from the retinohypothalamic tract (Ebling, 1996), and indirectly via retinally‐innervated cells of the intergeniculate leaflet of the thalamus (Harrington, 1997).

The intrinsic circadian rhythmicity of SCN neurons is readily observed *in vitro*: cultured SCN tissue displays rhythmic expression of a set of ‘clock genes’, the protein products of which act as transcription factors, accompanied by circadian rhythms of electrical discharge (Green & Gillette, 1982; Groos & Hendriks, 1982; Shibata *et al*. 1982; Abe *et al*. 2002). It is unclear exactly how the rhythms of electrical activity arise from the rhythms of gene expression. Circadian‐regulated genes may regulate the intrinsic electrophysiological properties of SCN neurones (Itri *et al*. 2005; Belle *et al*. 2009) and there is also evidence of circadian modulation of receptor expression, including receptors to glutamate, the principal neurotransmitter of the retinohypothalamic tract (Wang *et al*. 2008; Bendova *et al*. 2009).

Electrophysiological studies of the SCN began with *in vivo* studies in urethane‐anaesthetized rodents; these showed that the main effect of light is to increase neuronal discharge, consistent with neuroanatomical findings that retinal inputs form mostly excitatory contacts with cells of the SCN (Meijer & Rietveld, 1989). These electrophysiological studies consistently indicated that responses of SCN to light are stronger at night than during the day. However, there are also many cells that are inhibited by light, and many that are apparently unresponsive. Generally, it has been reported that neurones activated by light outnumber inhibited neurones by ∼2:1, as found by Groos & Mason (1980) and Jiao *et al* (1999), whereas many more neurones in the SCN may be unresponsive to acute changes in light (Saeb‐Parsy & Dyball, 2003; Drouyer *et al*. 2007; Brown *et al*. 2011; Walmsley & Brown, 2015).

A significant technical advance came through multi‐unit recording studies in freely moving rodents. These showed that the SCN displays higher activity during the subjective day than at subjective night (Inouye & Kawamura, 1979). Most studies using this approach did not resolve single unit activity but, in urethane‐anaesthetized mice, Brown *et al*. (2011) resolved single unit activities of light‐responsive cells, and reported that, for the most abundant subset of responsive neurones (35 cells that showed relatively sustained activation in response to light), there was a clear circadian rhythm of both spontaneous activity and light responsiveness, with highest activity during the subjective day and greatest responsiveness during subjective night. Smaller samples of neurones with a transient response and of neurones that were suppressed by light showed generally lower activity during the day than at night, and cells insensitive to light displayed a crepuscular pattern of basal activity, firing faster around dawn and dusk and more slowly around midday, a pattern similar to that ascribed to SCN neurons expressing high levels of Per1 (Belle *et al*. 2009). Thus, there is heterogeneity in circadian activity in SCN neurones, and this appears to reflect functionally disparate subpopulations of SCN neurones.

The rat SCN contains several neuronal types that can be distinguished biochemically. Most neurons express GABA and glutamate decarboxylase, a key enzyme of GABA synthesis (Moore & Speh, 1993; Belenky *et al*. 1996; Belenky *et al*. 2008), and GABA is considered to have an important role in intra‐SCN network activity and synchronization of firing rhythms among SCN neurons (Liu & Reppert, 2000; Shirakawa *et al*. 2000; Albus *et al*. 2005). Most of the synapses within the SCN are GABAergic (van den Pol, 1986), and both GABA_A_ and GABA_B_ receptor subtypes are expressed throughout the SCN (Gao *et al*. 1995; Belenky *et al*. 2003; Belenky *et al*. 2008). However, different subpopulations of SCN neurones express different neuropeptides (Ibata *et al*. 1999). The first subpopulation to be recognized was a large population of vasopressin neurones mainly in the dorsomedial SCN that provide a major output to other parts of the hypothalamus. Other substantial populations in this region (in the rat) express somatostatin and substance P. In the ventrolateral SCN, where most retinal afferents terminate, many neurones express vasoactive intestinal peptide, whereas many others express gastrin‐releasing peptide. There is also considerable functional heterogeneity that may not wholly correlate with biochemical heterogeneity Thus, it has been inferred that differentially timed waves of vasopressin, GABA and glutamate release from SCN efferents regulate preautonomic and neuroendocrine target neurons (Kalsbeek *et al*. 2006).

Alongside this biochemical diversity is a diversity of electrophysiological phenotype. A variety of spontaneous firing patterns have been reported in studies of the SCN *in vivo* and *in vitro*, including regularly firing neurons, neurons with a harmonic distribution of interspike intervals (ISIs) and isoperiodic bursting neurons, both *in vitro* (Groos & Hendriks, 1979; Walsh *et al*. 1992; Zhang *et al*. 1995; Pennartz *et al*. 1998; Brown *et al*. 2008) and *in vivo* (Aggelopoulos & Meissl, 2000; Saeb‐Parsy & Dyball, 2003; Sakai, 2014). In other regions of the hypothalamus, functionally or biochemically identified subpopulations of neurons display divergent electrophysiological phenotypes that can be well characterized by statistical features of their discharge activity, and, as would be expected, these phenotypes reflect differences in their intrinsic membrane properties.

The present study aimed to test whether light responsive neurons in the rat SCN display an electrophysiological phenotype that distinguishes them from non‐responsive cells, and also whether this phenotype is affected by circadian rhythms. To this end, we recorded from light‐responsive neurons in the rat SCN in the subjective day and in the subjective night, and characterized their spontaneous activity by statistical analysis of ISI distributions and higher‐order spike patterning.

## Methods

### Animals

All experiments were performed on rats under deep terminal anaesthesia in accordance with a UK Home Office project license reviewed by the University of Edinburgh Ethics Committee. One hundred and seventy‐two male Sprague–Dawley rats with a body weight of 250–450 g were used. They were housed under a 12:12 h light/dark cycle with food and water available *ad libitum*. Rats were kept under two different light regimes: a normal light cycle (lights on 07.00 h) or a reversed cycle (lights on 19.00 h); rats were maintained in these lighting conditions for at least 2 weeks before experiments. The eyes of the latter group were covered with foil until the beginning of recording, and experiments were conducted in darkened conditions: the experimental room was an internal room without windows, and the rig was surrounded by a blackout curtain to avoid interference by light entry from door opening. With the room lights off, the only light was from the recording displays, and, at the animal's eyes, the average light intensity was <1 lux. With room lights on, the intensity at the eyes was ∼100 lux. At the end of each experiment, rats were killed with a urethane overdose.

### *In vivo* extracellular recording

**Figure 1:**
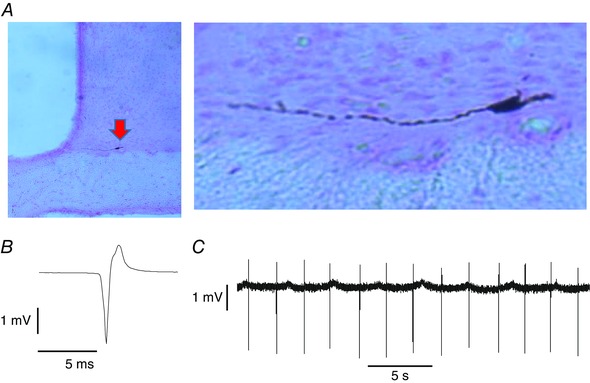
**Juxtacellularly labelled neurone in the SCN** *A*, juxtacellularly labelled neurone (arrowed) in the SCN, shown at higher magnification on the right. *B*, average action potential from the labelled neurone (average of 1000 spikes). The cell was recorded during the subjective day. *C*, extract of voltage trace showing individual spikes. Note the regularity of discharge. OC, optic chiasm; 3V, third ventricle.

Rats were anaesthetized with urethane (ethyl carbamate, 1.3 g kg^−1^ i.p.) and then tracheotomized, and the SCN was exposed by ventral surgery (Cui *et al*. 1997). During surgery, the eyes were covered with aluminium foil. With the rat prone in a stereotaxic frame, the mouth was gaped open to allow access to the roof of the mouth, and soft palate tissue was removed by cautery to expose the sphenoid bones. A small hole was drilled on the line of the suture between the basisphenoid and presphenoid bone, exposing the optic chiasm. The dura was nicked with a fine needle close to the midline and, under visual control, a glass microelectrode (tip diameter ∼1 μm) was lowered into the tissue (between bregma −0.92 mm to −1.4 mm; lateral 0.1 mm to 0.4 mm; Paxinos & Watson, 2006). Single neurons were recorded using conventional extracellular recording techniques (Sabatier *et al*. 2004; Sabatier & Leng, 2010; Leng *et al*. 2014). Recordings were made at a depth of 400–1500 μm from the first contact of the electrode with the ventral surface of the optic chiasm. This consistently led the electrode tip into the SCN, as confirmed histologically in pilot experiments using electrodes filled with 2% pontamine sky blue in 0.5 m sodium acetate: in these experiments, dye was ejected by applying 10 V for 10 min through the electrode. In subsequent experiments, we attempted to label cells juxtacellularly using electrodes filled with 1.5% *N*‐(2 aminoethyl) biotinamide hydrochloride (neurobiotin; Vector Laboratories, Peterborough, UK) in 0.5 m NaCl (Pinault, 1996), visualizing labelled cells with 3,3′‐diaminobenzidine stain. However, although we could label light‐unresponsive cells in the SCN, attempts to label light‐responsive cells were unsuccessful. No light‐responsive cells were labelled in more than 100 attempts (several attempts were made in each experiment). By contrast, in 12 attempts to label unresponsive cells, six cells were labelled, all clearly within the SCN (Fig. 1). We eventually abandoned these attempts and used electrodes filled with 0.9% NaCl.

The spontaneous activity of each neurone was recorded for at least 5 min using an Axopatch 200B amplifier (Axon Instruments, Burlingame, CA, USA) or a microelectrode AC amplifier (Model 1800) (A‐M systems; Sequim, WA, USA), a digital interface and Spike2 software (Cambridge Electronic Design). Light stimulation (5 s light‐on, 100 lux, off, <1 lux; measured at the eyes) was applied to both eyes or to the contralateral eye to identify cells as light responsive. To study the effect on firing patterns, we alternated bright (∼100 lux for 200 s) and dark conditions under (<1 lux) and averaged the recorded data for each bright or dark phase.

### Statistical analysis

ISI histograms, auto‐ and cross‐correlograms and waveform averages were calculated using Spike2 software, and the data were exported to Excel 2010 (Microsoft Corp., Redmond, WA, USA) for further analysis. We constructed hazard functions (Sabatier *et al*. 2004; Leng *et al*. 2014) in 1 ms bins using the formula [hazard in bin (*t*, *t* + 1)] = [number of ISIs in bin (*t*, *t* + 1)]/(number of ISIs of length > *t*). The hazard function displays how the excitability of a cell changes with the time subsequent to the last spike, plotting the incidence of spikes as a proportion of the size of the residual tail of the ISI distribution. Thus, for a given cell, the hazard of a spike occurring, for example, between 30 and 31 ms after a spike is calculated as the total number of spikes that arise with an ISI of between 30 and 31 ms divided by the total number of spikes that arise with an ISI of greater than 30 ms (i.e. the hazard function reports the probability of a spike occurring in each interval). When ISI data are plotted this way, a negative exponential distribution (the distribution characteristic of random events) becomes a constant ‘hazard’ proportional to the average firing rate. Deviations from this constant level then become interpretable as periods of increased or decreased excitability. For subpopulations, consensus functions were calculated from the means of hazard functions. Cells were classed as ‘harmonic’ when the ISI distributions showed three or more modes separated by a similar interval.

### Carbenoxolone

In six experiments, we tested the effects of i.c.v. administration of the gap junction blocker carbenoxolone (carbenoxolone disodium; Sigma, St Louis, MO, USA) on the activity of an SCN cell. Carbenoxolone (2 μl of 10 or 100 mg ml^−1^ solution in artificial cerebrospinal fluid) was administered into the third ventricle over 2–3 min via a cannula inserted into the ventricle through the median eminence, 3 mm caudal to the recording site. These experiments were all conducted in rats maintained on a normal light cycle.

### Open data

Extended documentation of the data from the present study is provided in the Supporting information (Data S1).

## Results

### General observations

**Figure 2:**
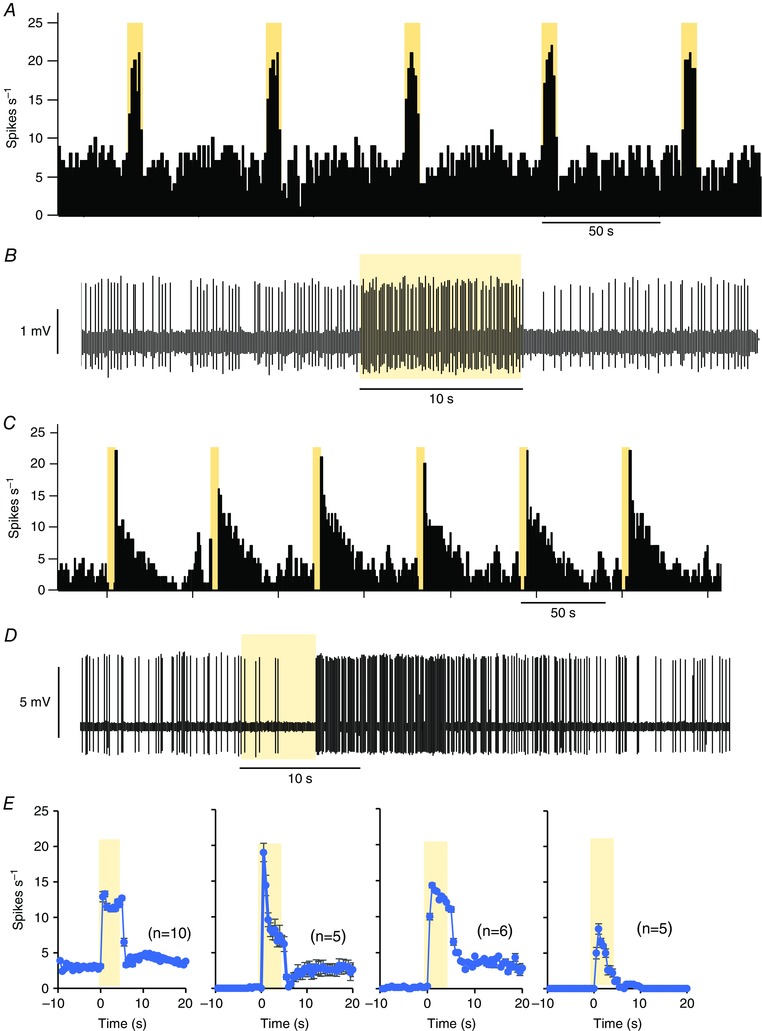
**Light‐on cells in the SCN** *A*, responses to light of a light‐on cell in the SCN (firing rate in 1 s bins). Note the consistency of responses evoked by 5 s exposures to light (yellow bars). *B*, extract of voltage trace showing individual spikes and response to a light pulse. *C*, responses to light of a light‐off cell. *D*, extract of voltage trace showing individual spikes. *E*, Mean responses to 5 s pulses of light in four light‐on cells recorded during subjective day, showing diverse response profiles (mean ± SEM of responses to *n* pulses, as indicated in parentheses).

We recorded from SCN cells between zeitgeber time (ZT)4 and ZT10 in 66 rats under normal lighting (12 h on from 07.00 h; 12 h off) and between ZT16 and ZT22 in 53 rats under reversed lighting (12 h on from 19.00 h; 12 h off). The eyes of the latter group were covered with foil until the beginning of recording, and experiments were conducted in darkened conditions. We recorded the spontaneous activity of each cell for >5 min, and then tested its response to 5 s light at 1500 lux (Fig. 2 *A–D*).

Light responsiveness was complex, and response patterns were diverse (Fig. 2 *E*): we classified cells as light‐on cells when they increased their firing rate in response to 5 s of exposure to light, or decreased it in the first 5 s in response to darkness; as light‐off cells when they decreased their firing frequency in response to light or immediately increased it in response to darkness; and as non‐responsive cells. We then recorded most light‐responsive cells in alternating bright (100 lux for >200 s) and dark (<1 lux for >200 s) conditions (Fig. 2).

Light‐on cells were found more often than light‐off cells at both recording times. Of 222 light‐responsive cells recorded between ZT4 and ZT10, 150 (68%) were light‐on cells and 72 (32%) were light‐off cells. Of 184 light‐responsive cells recorded between ZT16 and ZT22, 125 (68%) were light‐on cells and 59 (32%) were light‐off cells.

Light‐responsive cells were recorded at depths of up to 1.5 mm from the first contact of the electrode with the optic chiasm, although most (88%) were found between 200 and 900 μm (mean (SEM) 663 ± 15 μm, *n* = 249). There was no apparent difference in the depths at which light‐on and light‐off cells were encountered, and no relationship between the recording depth and mean firing rate or observed discharge pattern (data not shown).

Individual cells were readily discriminated above the background noise level when mean spike heights exceeded 0.5 mV, and, in many cells, mean spike heights were 5 mV or more. Spike heights varied with movements of the electrode, and varied slowly over the course of most recordings, and this were not generally considered meaningful. No changes in spike height were seen in response to either short or prolonged light stimulation, although most light‐responsive SCN cells fired occasionally with very short ISIs of <5 ms and, in these cases, the second spike of such doublets was consistently smaller than the first (see Supporting information, Data S1, sheet ‘Light‐on example’). Spike waveforms varied between cells, although we saw no consistent association of waveform with response type (see Supporting information, Data S1, sheets ‘Light‐on spikes’ and ‘Light‐off spikes’).

### Light‐on cells

**Figure 3:**
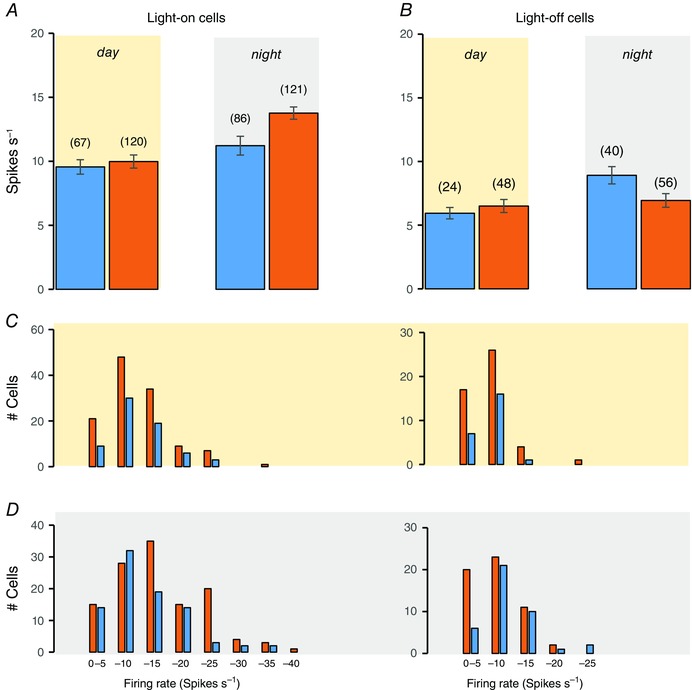
**Firing rates of light‐responsive cells** Mean ± SEM firing rates of light‐on cells (*A*) and light‐off cells (*B*) recorded in the subjective day (yellow background) or night (grey background) with light‐on (orange bars) or off (blue bars). Numbers of cells are given in parentheses. *C*, distributions of firing rates for cells recorded in the subjective day. *D*, distributions of firing rates for cells recorded in the subjective night. Light‐on cells are shown on the left and light‐off cells on the right; cells recorded with light‐on are shown as orange bars and cells recorded with light‐off are shown as blue bars.

During the subjective day (ZT4 to ZT10), light‐on cells recorded in bright conditions fired at 10.0 ± 0.5 spikes s^−1^ (mean ± SEM; range 0.5–32.1; *n* = 120). During the subjective night (ZT16 to ZT22), light‐on cells recorded in the dark fired at (11.2 ± 0.7 spikes s^−1^; range 0.9–33.3; *n* = 88), which is not significantly different from light‐on cells recorded in bright conditions during the subjective day (*P* = 0.34, Mann–Whitney *U* test) (Fig. 3).

**Figure 4:**
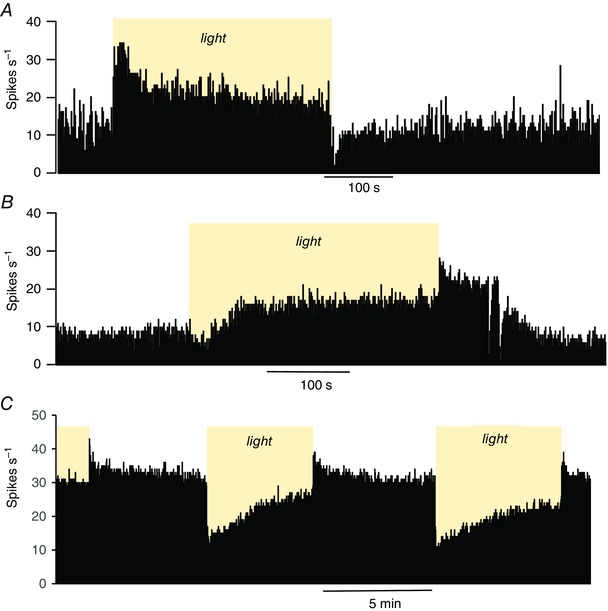
**Responses of SCN neurones to 200 s exposure to light (yellow regions, 100 lux)** The plots are of firing rate in 1 s bins. *A*, this cell is strongly activated at the onset of light; the firing rate adapts during continued exposure, and falls promptly when the light is switched off. *B*, this light‐off cell responds to light with an initial fall in firing rate but then speeds up to exceed the initial firing rate. When the light is switched off, the firing rate increases further before falling again to the initial firing rate. *C*, this light‐off cell shows a simple adapting inhibition in response to light.

Sixty‐three light‐on cells recorded in the subjective day and 84 light‐on cells recorded in subjective night were tested with 200 s exposures to light separated by similarly periods of dark (Fig. 4). In both groups, some cells (16/63 in subjective day; 14/84 in subjective night) showed inhibition after initial activation; thus, some fired more slowly during sustained exposure to light than during sustained exposure to dark. Overall, the 63 cells recorded in the subjective day fired at 9.6 ± 0.6 spikes s^−1^ in the dark and at 11.4 ± 0.7 spikes s^−1^ in the light (difference 1.8 ± 0.5 spikes s^−1^). The 84 cells recorded at subjective night fired at 10.9 ± 0.8 spikes s^−1^ in the dark and at 14.9 ± 0.9 spikes s^−1^ in the light (difference 4.1 ± 0.6 spikes s^−1^, which is significantly different from cells recorded in the subjective day, *P = *0.003, Mann–Whitney *U* test).

Thus, light‐on cells were more active when recorded in the dark during the subjective night than when recorded in the light during the subjective day, and were more strongly activated by light during the subjective night.

### Light‐off cells

During the subjective day, light‐off cells fired at 6.5 ± 0.4 spikes s^−1^ (range 1.4–20.3, *n* = 48) in the light and, during the subjective night, at 8.9 ± 0.4 spikes s^−1^ (range 2.3–24.4; *n* = 40) in the dark (Fig. 3 *B*). Again, some cells were tested with repeated prolonged exposure to light. Of these, 23 cells recorded in the subjective day fired at 5.9 ± 0.5 spikes s^−1^ in the dark and at 6.2 ± 0.5 spikes s^−1^ in the light (difference +0.2 ± 0.4 spikes s^−1^); in 14 of these cells, the initial activation by dark was not sustained, and was followed by inhibition. By contrast, 37 light‐off cells recorded in the subjective night fired at 8.8 ± 0.7 spikes s^−1^ in the dark and at 7.1 ± 0.6 spikes s^−1^ in the light (difference −1.7 ± 0.4 spikes s^−1^, which is significantly different from cells recorded in the subjective day, *P = *0.002, Mann–Whitney *U* test) and most (24/37) were activated throughout 200 s of exposure to dark.

Thus, in all conditions, light‐off cells were less active than light‐on cells. Light‐off cells were more active in the dark during the subjective night than in the light during the subjective day, and were more responsive to changes in light during the subjective night.

### Spontaneous firing patterns of SCN cells

We analysed the spontaneous firing patterns of SCN cells by constructing ISI distributions, hazard functions and autocorrelograms (see examples in the Supporting information, Data S1). Most cells that did not show a clear response to light had a unimodal distribution of ISIs. Of 177 non‐responsive cells, 12 cells with a mean ± SEM rate of 11.4 ± 3.7 (range 2.5–43.1) spikes s^−1^ had a symmetrical distribution of ISIs around a single mode, the value of which was inversely related to the mean ± SEM firing rate; 40 cells [5.8 ± 0.7 (range 0.9–20.6) spikes s^−1^] had a skewed ISI distribution with a single mode at >50 ms and few ISIs shorter than the mode, indicating a prolonged post‐spike refractoriness; 41 cells [7.8 ± 1.3 (range 0.5–38.8) spikes s^−1^] showed a post‐spike hyperexcitability giving a prominent peak in hazard functions at 40 ± 7 (range 10–190) ms. Another 69 cells fired too slowly (<2 spikes s^−1^) and irregularly to classify clearly, and five cells (4 ± 1.5 spikes s^−1^) displayed a multimodal ISI distribution similar to the light‐responsive cells described below (Supporting information, Data S1, ‘Non‐responsive cells’).

Because only six cells were labelled juxtacellularly, we cannot be sure that all were in the SCN. The depths at which these cells were recorded overlapped with the depths at which light‐responsive cells were recorded, and many were found amongst light‐responsive cells, although some electrode tracks may have passed through the margins of the SCN encountering cells that it would be inappropriate to describe as SCN cells. Accordingly, we do not consider these cells further and note that not all cells within the SCN are light‐responsive, and also that non‐responsive cells in or close to the SCN have heterogeneous electrophysiological phenotypes, different from the phenotypes of light‐responsive neurones that we describe below.

**Figure 5:**
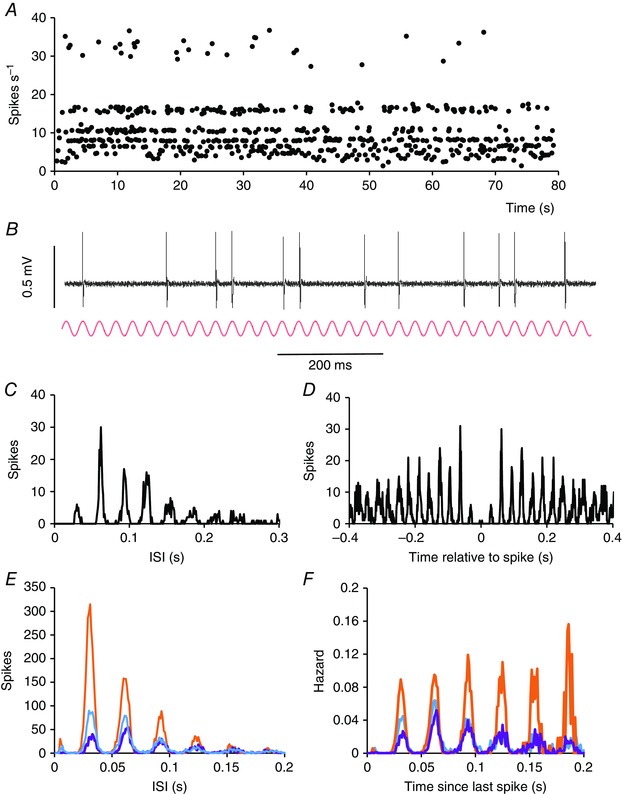
**Harmonic discharge patterning** *A*, Instantaneous frequency plot of a representative harmonic SCN cell recorded during the subjective day in the light. Each point that is plotted is the reciprocal of the preceding ISI, plotted at the time of spike occurrence. Note the conspicuous ‘banding’ of instantaneous frequencies. This cell was a light‐off cell (not shown) and its average firing rate over the period shown was 7.6 spikes s^−1^. *B*, sample of voltage record from the cell shown in (*A*), inverted to show spikes as positive‐going, above a sine wave (red) with a frequency of 32 Hz. Note that spikes are coincident with peaks of the sine wave. *C*, ISI distribution for the cell shown in (*A*). The distribution is multimodal, with the modes corresponding to multiples of 31ms: harmonics of a fundamental frequency of 32 Hz. *D*, autocorrelogram of the cell shown in (*A*). Peaks occur at intervals of 32 ms. *E*, ISI distributions from three different SCN cells. The cell in orange was recorded at subjective night and fired at 17 spikes s^−1^, the cell in blue was also recorded at subjective night and fired at 8.6 spikes s^−1^, and the cell in purple was recorded during the subjective day and fired at 5.6 spikes s^−1^. Despite the differences in firing rates, the three cells all have a fundamental frequency of 32 Hz. *F*, hazard functions corresponding to the ISI distributions in (*E*).

Most light‐responsive cells had ISI distributions with multiple modes separated by a common interval: harmonics of a fundamental frequency (Fig. 5). When the discharge activity of these ‘harmonic cells’ was displayed as instantaneous frequency plots (i.e. when the arrival time of each spike was plotted against the inverse of the preceding ISI), the plots showed clear bands corresponding to these harmonics (Fig. 5 *A*). These bands correspond to peaks in the ISI distribution (Fig. 5 *C*) and to peaks in the autocorrelogram (Fig. 5 *D*), although multiple peaks in the autocorrelogram (but not in the ISI distribution) also arise for regularly firing cells that are not light responsive. Different harmonic cells firing at very different mean firing rates had multimodal ISI distributions that displayed the same fundamental frequency (Fig. 5 *E*), and hazard functions (Fig. 5 *F*) showed that the rhythmic excitability is sustained over multiple cycles with little if any decay.

**Figure 6:**
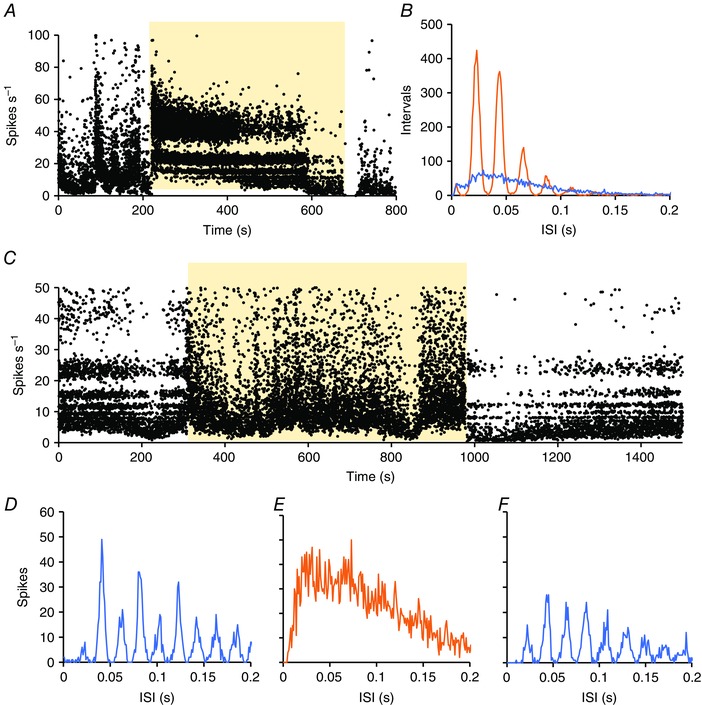
**Effects of light exposure on harmonic discharge patterning in SCN neurones** *A*. Instantaneous frequency plot of an SCN neurone activated by light (yellow background). In this cell, activity in the dark was irregular and devoid of any harmonic patterning of ISIs. During exposure to light however, the activity immediately changed to a strongly harmonic pattern, as indicated by the “banding” of spike frequencies. *B*. ISI distributions of 500s of activity in the light (orange) and of 500s of activity in the dark (blue). *C*, Instantaneous frequency plot of a second SCN neurone activated by light (yellow background). In this cell, activity in the dark was strongly harmonic patterning as indicated by the “banding” of spike frequencies. During exposure to light however, the activity immediately changed to a non‐harmonic pattern, *D‐F*, ISI distributions of 500s of activity in the dark (*D*, blue) of 500s of activity in the light (*E*, orange) and of 500s after return to dark (*F*, blue).

Most light‐responsive cells displayed these features [76% (79/104) of light‐off cells and 91% (215/237) of light‐on cells] during at least part of the recording: conversely, almost all harmonic cells in the SCN (294/299) were acutely responsive to light. In a few cells, harmonic patterning occurred only during exposure to light or to dark (Fig. 6). Switching the light‐on induced harmonic patterning in five light‐on and in seven light‐off cells that showed no clear harmonic patterning in the dark, whereas switching the light‐off induced harmonic patterning in five light‐on cells and in five light‐off cells that showed no such patterning when the light was on.

**Figure 7:**
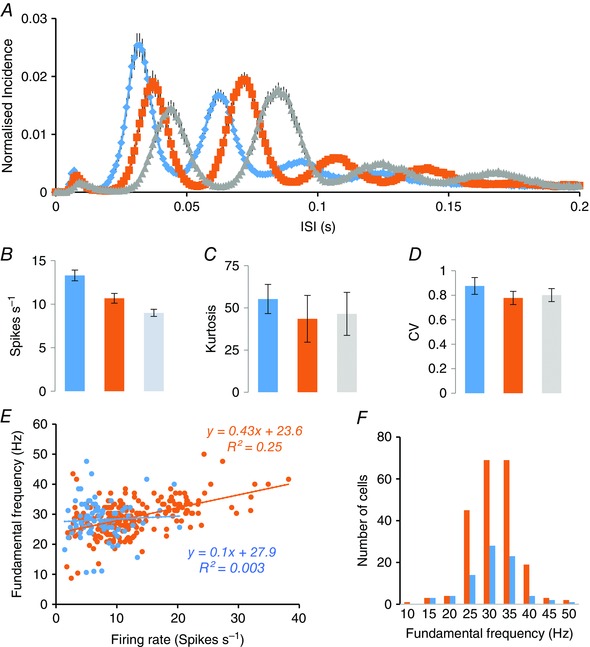
**Statistical characteristics of discharge patterning in light‐responsive cells in the SCN** *A*, ISI distributions (mean ± SEM of distributions normalized to total number of ISIs) of harmonic SCN cells with fundamental frequencies of 50–29 Hz (blue; n = 118), 29–25 Hz (orange; n = 75) and 26–16 Hz (grey; *n* = 89). All distributions were constructed from 200 s of spontaneous activity recorded in bright conditions. *B*, firing rates (mean ± SEM) of the three subsets of harmonic cells. *C*, kurtosis (mean ± SEM) of the ISI distributions in (*A*). *D*, coefficient of variation of ISIs for the three subsets. *E*, relationship of fundamental frequency and mean firing rate. For each of 215 harmonic light‐on cells (orange symbols) and 79 light‐off cells (blue symbols), the fundamental frequency is plotted against the mean firing rate, with linear regression lines indicated. *F*, distributions of fundamental frequencies for the light‐on cells (orange) and the light‐off cells (blue).

For each harmonic cell, we measured the interval between modes to define the ‘fundamental frequency’ of their discharge. Figure 6 summarizes the discharge characteristics separated into three groups containing similar numbers of cells, according to the fundamental frequency (50–30 Hz; 30–27 Hz, 27–20 Hz; ISI distributions) (Fig. 6 *A*). For harmonic cells as a whole, there was a weak inverse relationship between fundamental frequency and mean firing rate (Fig. 7 *B*), although no difference between subgroups in either kurtosis of the ISI distribution (Fig. 7 *C*) or coefficient of variation of ISIs (Fig. 7 *D*). The relationship between fundamental frequency and firing rate was significant for light‐on cells only (Fig. 7 *E*).

In bright conditions, harmonic light‐on cells fired with a mean ± SD fundamental frequency of 28.9 ± 5.8 Hz (*n* = 215), which is very close to that of light‐off cells (28.1 ± 6.5 Hz; *n* = 79) and the distribution of fundamental frequencies was similar for light‐on cells and light‐off cells (Fig. 7 *F*). There were no significant differences in fundamental frequency for either group between subjective day and subjective night. Cells recorded close together tended to have similar fundamental frequencies, and much of the population variance reflected variation between experiments rather than variation within experiments. To quantify this, we analysed 59 experiments in which more than one harmonic cell had been recorded, and calculated the intra‐animal and inter‐animal variation (for full data, see the Supporting information, Data S1). In this data set (184 cells), the mean ± SD fundamental frequency (mean of animal means; *n* = 59) was 29 ± 4.2 Hz, whereas the SD within animals was just 2.8 Hz (124 d.f.). There was also a small but significant tendency for the fundamental frequency of harmonic cells to decline over time during experiments regardless of whether they were conducted in normal or reversed light conditions (not shown). Thus, the observations indicated that, at any given time, most light‐responsive cells in the SCN fired in a pattern locked to an oscillatory rhythm with a frequency close to 30 Hz.

**Figure 8:**
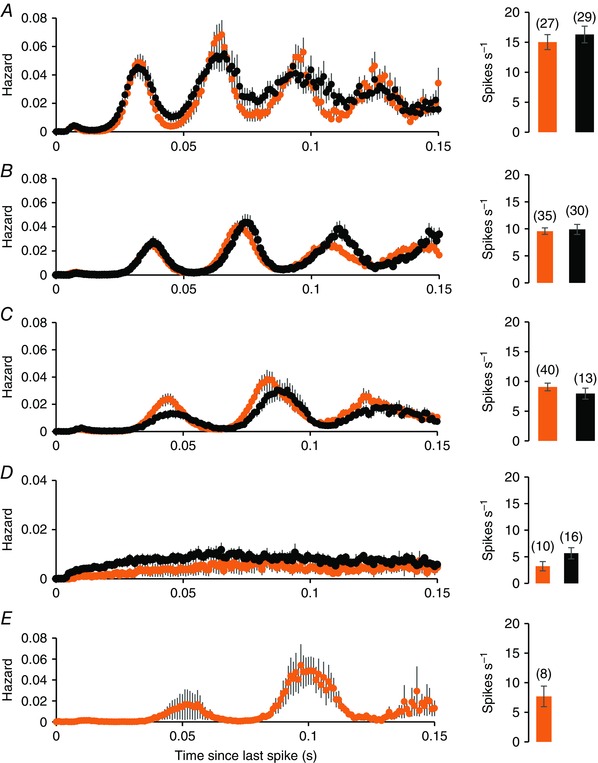
**Hazard functions of light‐on cells recorded in the subjective day in bright light (orange) and light‐on cells recorded in the subjective night in dark conditions (black)** *A*, *B* and *C*, harmonic cells sorted by fundamental frequency: 50–29 Hz (*A*), 29–25 Hz (*B*) and 26–16 Hz (*C*). *D*, hazard functions of non‐harmonic cells, and (*E*) shows hazard functions of a subgroup of cells recorded in the subjective day with an unusually long fundamental frequency. For each group, the plots are mean ± SEM hazards. To the right of the hazard functions, the bars show the mean ± SEM firing rates of the subgroups, with the number of cells in each subgroup in parentheses. There is no significant difference in the mean firing rate of light‐on cells between the two conditions.

**Figure 9:**
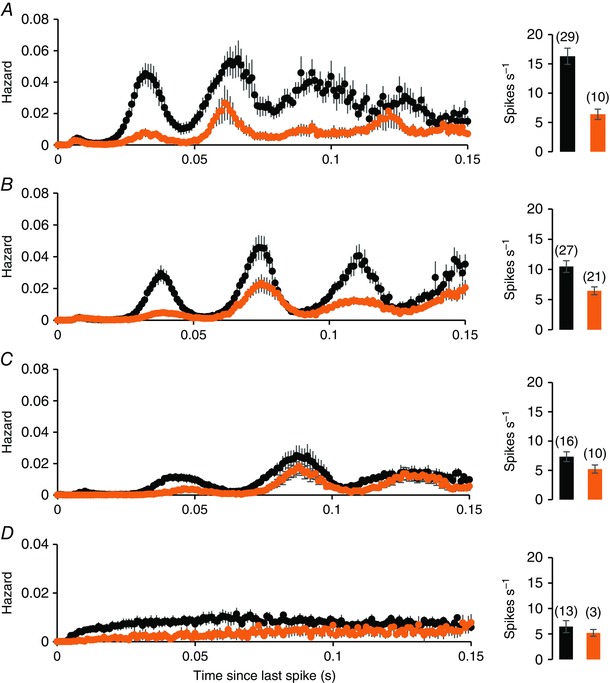
**Hazard functions of light‐off cells recorded in the subjective day in bright light (orange) and light‐off cells recorded in the subjective night in dark conditions (black)** *A*–*C*, subgroups sorted by fundamental frequency: 50–29 Hz (*A*), 29–25 Hz (*B*) and 26–16 Hz (*C*). *D*, hazard functions of non‐harmonic light‐off cells. For each group, the plots are mean ± SEM hazards. To the right of the hazard functions, the bars show the mean ± SEM firing rates of the subgroups, with the number of cells in each subgroup in parentheses. Light‐off cells, in all subgroups, are more active in subjective dark than in subjective day.

To understand how discharge patterning changes through a normal day, we compared cells recorded in bright conditions during the subjective day with cells recorded in the dark during the subjective night. From the ISI distributions, we calculated hazard functions because these display the harmonic patterning more appropriately, and we grouped harmonic cells by fundamental frequency as above. For light‐on cells (Fig. 8), there was little difference in spontaneous discharge patterning between subjective day and night. All subgroups of light‐off cells (Fig. 9) were more active in subjective night, including non‐harmonic cells (Fig. 9 *D*).

**Figure 10:**
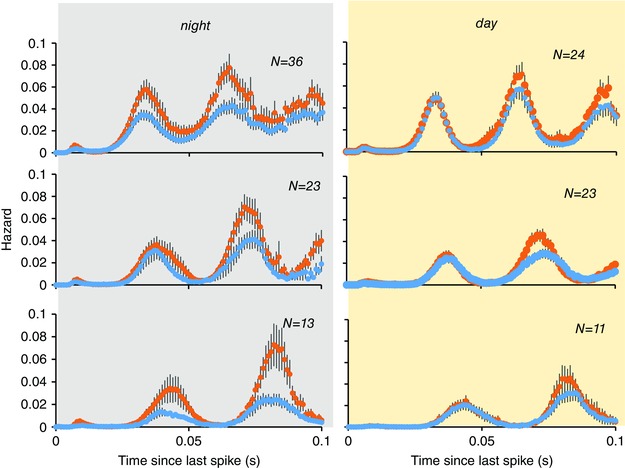
**Hazard functions (mean ± SE) of harmonic light‐on cells analysed with light‐on (orange) and light‐off (blue)** Cells were grouped by fundamental frequency (top; middle; bottom). Exposure to light had no effect on the fundamental frequency in any group. Light‐on cells recorded at subjective night (grey background) were much more responsive to light than cells recorded during subjective day (yellow background).

**Figure 11:**
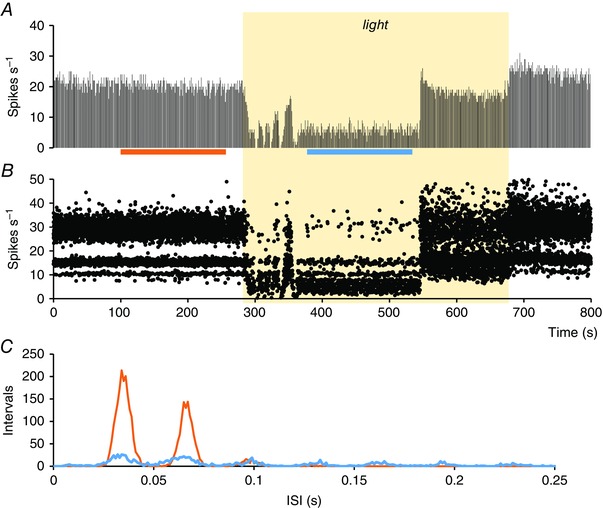
**Effects of light on discharge patterning in a light‐off cell in the SCN** *A*, firing rate (in 1 s bins) of an SCN cell recorded during the subjective day that was inhibited by exposure to light (yellow shaded bar). *B*, instantaneous frequency plot (cropped at 50 Hz) corresponding to the rate record in (*A*), showing the ‘banding’ characteristic of a harmonic discharge pattern. *C*, ISI distributions (mean ± SEM) constructed from the 150 s periods indicated by the orange and blue bars in (*A*). This cell has a fundamental frequency of ∼30 Hz, and this is the same in the light as in the dark.

Although the discharge activity of light‐on cells at night in the dark was similar to that during the day in the light, there was a difference in their responsiveness to light. We constructed hazard functions for 72 light‐on cells at subjective night and 58 cells during the day in both bright and dark conditions. The cells were grouped by their fundamental frequency, as described above. For cells recorded during the day, the hazard functions showed little difference between light and dark conditions. At subjective night, although cells were much more active in bright conditions than in the dark (Fig. 10), exposure to light had no effect on the fundamental frequency of their discharge. This was true also for light‐off cells (an example is provided in Fig. 11; full data are provided in the Supporting information, Data S1).

### Paired recordings

Thus, most light‐responsive cells in the SCN fired in a pattern locked to an oscillatory rhythm with a frequency of close to 30 Hz. To investigate synchronicity between adjacent cells, we analysed six paired recordings of two harmonic cells, four of non‐harmonic cells, and one example of a harmonic cell with a regular cell, where the difference in spike heights was sufficiently large that both could be reliably discriminated (Fig. 11 *A*).

**Figure 12:**
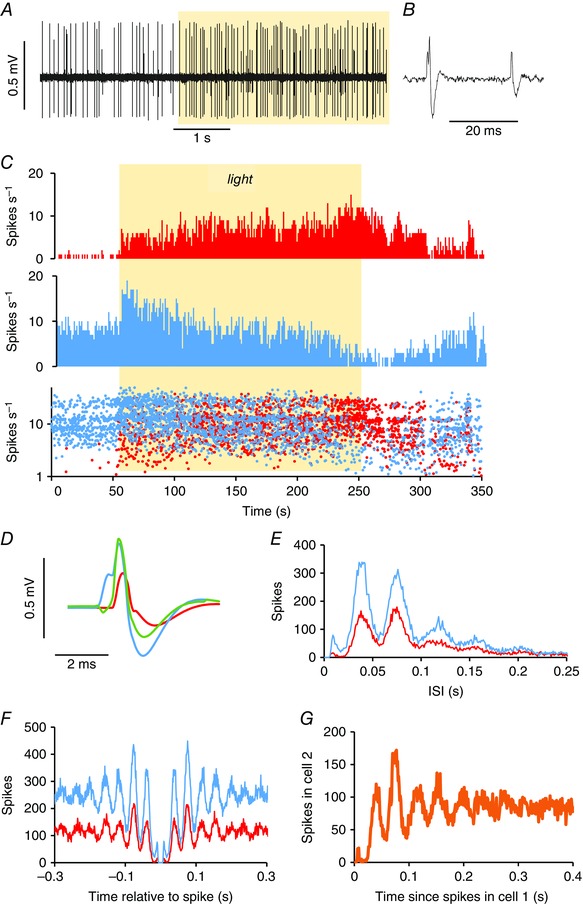
**Simultaneous recording of two harmonic cells in the SCN** *A*, extract of voltage trace from a recording of two cells through a single electrode, with spike heights sufficienty different to allow clear separation; the record is inverted to show spikes as positive‐going. *B*, section at higher resolution, note that the larger spike has a complex waveform; the rising phase has an element that appears identical with the waveform of the smaller spike. *C*, firing activity (in 1 s bins) of the larger spikes (blue) and the smaller spikes (red) above an instantaneous frequency plot (plotted on a log scale and cropped at 50 Hz) superimposing the recordings of the larger and smaller spikes. Both cells were activated by light (yellow shaded bar). *D*, average waveform of spikes in the larger cell (blue) and the smaller cell (red) (for the period shown in (*C*). The green waveform is the result of subtracting the waveform of the smaller cell (red), displaced by 0.6 μs, from that of the larger cell. This suggests that the apparent waveform of the larger cell comprises tightly coupled spikes from two cells. *E*, ISI distributions of the two spikes constructed from a 2520 s period in which the two spike heights could be reliably discriminated. The two cells both have a harmonic discharge pattern with an identical fundamental frequency. *F*, autocorrelograms of the two cells over the whole period of recording. *G*, cross‐correlogram of the activity in the two cells.

In each paired recording of harmonic cells, both cells were light‐on cells, and their autocorrelograms were multimodal, with a fundamental frequency close to 30 Hz. In each case, the activity of the two cells had identical fundamental frequencies, and cross‐correlograms indicated that these rhythms were strictly locked (Fig. 11). Thus, recognizing synchronous spiking depended on inferences from the spike waveform. In the case shown in Fig. 12, the waveform of spontaneous spikes of the bigger cell showed a notch just before the peak of the spike (Fig. 12 *D*, blue). The waveform of this notch matched the spike waveform of the smaller cell (Fig. 12 *D*, red) and subtracting the average spike waveform of the ‘smaller’ cell from that of the ‘bigger’ cell eliminated the notch entirely (Fig. 12 *D*, green). These results indicated that neighbouring harmonic cells discharge synchronously, with very tightly locked spikes.

**Figure 13:**
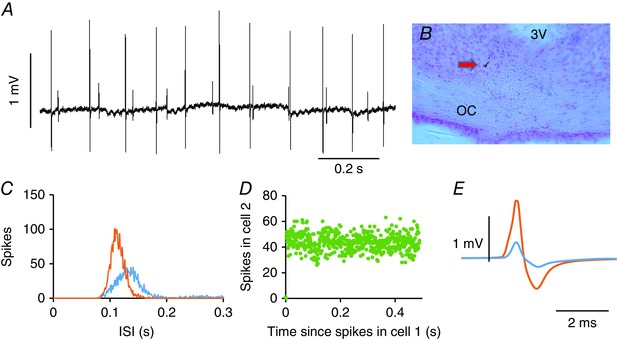
**Simultaneous recording of two regularly firing cells in the SCN** *A*, extract of voltage trace from a recording of two cells through a single electrode, with spike heights sufficiently different to allow clear separation; the record is inverted to show spikes as positive‐going. *B*, recording location in the SCN between the third ventricle (3 V) and the optic chiasm (OC). *C*, ISI distributions constructed from a 500 s period in which the two spike heights could be reliably discriminated. The two cells are both discharging regularly but at different periods. *D*, cross‐correlogram of the activity in the two cells, showing that they are firing independently. *E*, average waveforms of spikes in the larger cell (orange) and the smaller cell (blue).

We found only one example of a harmonic cell recorded with a non‐harmonic cell (not shown) and the cross‐correlogram indicated that these cells fired independently. We analysed four examples of paired recordings of non‐harmonic cells that were apparently within the SCN because they were obtained close to recordings of light‐responsive cells. Figure 13 shows the analysis of one pair, each of which fired regularly with unimodal ISI distributions (Fig. 13 *B*). The auto‐correlograms were multimodal but the periods differed (108 ms and 126 ms) and the cross‐correlograms indicated that these cells fired independently (Fig. 13 *C*). Similarly, each of the other three pairs of non‐harmonic cells all showed no indication of correlated discharge.

### Isoperiodic bursting

**Figure 14:**
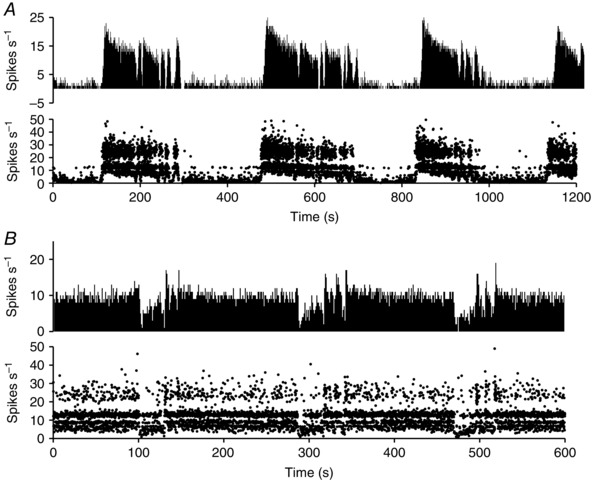
**Isoperiodic bursting in SCN cells** Examples of slow isoperiodic bursting in (*A*) a light‐on cell and (*B*) a light‐off cell. In the light‐on cell, the phase of high activity ends with a fractionation of the discharge pattern (type 1 bursting), whereas, in the light‐off cell, the phase of high activity begins with a fractionated pattern (type 2 bursting). For each cell, the firing rate (in 1 s) bins) is shown above the record of instantaneous firing rate (cropped at 50 Hz). The instantaneous firing rates show the ‘banding’ characteristic of harmonic discharge.

Some light‐responsive cells displayed spontaneous slow alternations in activity in stable light conditions (Fig. 14) and this was more common in cells recorded during subjective day (69 of 121 light‐on cells, 23 of 52 light‐off cells during subjective day; 26 of 125 light‐on cells and three of 58 light‐off cells during subjective night). We recognized two subtypes of periodic firing. In light‐on cells, the active phase usually ended with fractionated bursting (Fig. 14 *A*): 83 of 94 phasic light‐on cells displayed this ‘type 1’ pattern (62 cells during subjective day and 23 during subjective night) and just two of 26 phasic light‐off cells (both recorded during subjective day). By contrast, light‐off cells usually showed fractionated bursting at the beginning of the active phase (Fig. 14 *B*: 22 of 26 cells, 20 during subjective day and two during subjective night), although only two light‐on cells showed this ‘type 2’ pattern (both during subjective day). Other periodic cells showed a simple alternation between phases of high and low activity. We measured the period of alternations for cells in which we had a sufficiently long period of control activity to see a clear repeated cycle. Periodic light‐on cells with type 1 pattern had a mean (SEM) cycle length of 230 ± 18 s (*n* = 35) in subjective day and 236 ± 24 s (*n* = 15) in subjective night, whereas periodic light‐off cells in subjective day with a type 2 pattern had a mean (SEM) cycle length of 207 ± 31 s (*n* = 15).

### Carbenoxolone

**Figure 15:**
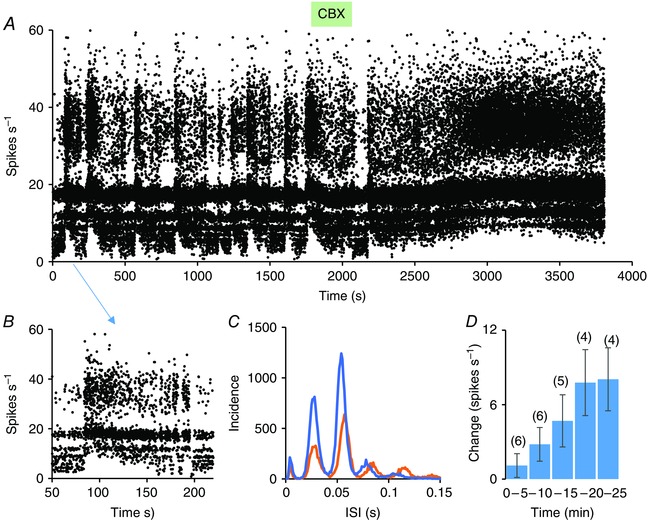
**Effects of carbanoxolone on harmonic SCN cells** *A*, instantaneous frequency plot (truncated at 60 spikes s^−1^) of the activity of a harmonic cell in the SCN in response to i.c.v. administration of 20 μg carbenoxolone (CBX, green bar). The cell was recorded in dark conditions, and showed isoperiodic type 1 bursting that was abolished after CBX; however, the harmonic discharge patterning persisted. *B*, expansion of the part of the record indicated by the blue arrow to show a single type 1 burst; note that the active period ends with fractionated bursting. *C*, ISI distributions from 1200 s in the control period (0–1200 s; orange) and after CBX (from 2600–3800 s). There is a small increase in the fundamental frequency of harmonic discharge in this cell. *D*, mean ± SEM change in firing rate of six harmonic SCN cells in 5 min bins after i.c.v. injection of CBX; the numbers in parentheses show the number of cells in each 5 min interval.

In six experiments, we tested the effects of i.c.v. administration of the gap junction blocker carbenoxolone on the discharge patterning of a harmonic SCN neurone (Fig. 15). Two of the tested cells showed isoperiodic bursting activity (Fig. 15 *B* and *C*). In all cells, harmonic activity persisted, with a small increase in fundamental frequency in two of the six cells (Fig. 15 *C*). Each of the six cells progressively increased in firing rate after carbenoxelone over the 20 min after injection (Fig. 15 *D*) and the bursting activity was abolished in each of the two bursting cells.

## Discussion

In the present study, we tested whether light responsive neurons in the rat SCN display an electrophysiological phenotype that distinguishes them from non‐responsive cells, and whether this phenotype is affected by circadian rhythms. We found that almost all light‐responsive cells in the rat SCN (294 of 299 cells) discharged with a multimodal harmonic distribution of ISIs in at least some conditions, whereas this discharge pattern was observed in very few SCN cells that were not light responsive (five of 177 cells). This harmonic pattern apparently reflects a subthreshold oscillatory rhythm in excitability at a frequency of close to 30Hz: the inferred fundamental frequency did not differ between light‐on cells and light‐off cells, and was generally unaffected by light exposure or by time of day. Light‐on cells were more active than light‐off cells both at night and during the day, and light‐off cells were much more active at night than during the day. We saw no other differences in their electrophysiological phenotype between day and night from analysis of their ISI distributions, although a subset of harmonic cells displayed slow isoperiodic bursting, and this was more common during subjective day than during subjective night.

Most previous studies of SCN neurons did not report ISI distributions systematically, although similar harmonic distributions were reported for 61 light‐responsive SCN cells by Aggelopoulos & Meissl (2000) in rats anaesthetized with either urethane or pentobarbitone, and in a few SCN cells reported by Saeb‐Parsy & Dyball (2003) who recorded from many SCN cells in urethane‐anaesthetized rats but who tested few of these with light. Similar harmonic distributions were also observed in some SCN cells recorded in hypothalamic slices *in vitro* by Zhang *et al*. (1995) and by Brown *et al*. (2008). In our recordings from other regions of the rat hypothalamus, we have never seen harmonic discharge patterns in the supraoptic nucleus, although we have occasionally seen an apparently similar harmonic patterning in a very few cells in the perinuclear zone dorsolateral to that nucleus (G. Leng, unpublished observations). We have never seen a harmonic pattern in the arcuate nucleus (Honda *et al*. 1999); in the ventromedial nucleus, a substantial subpopulation of neurones discharge harmonically but with a very much lower fundamental frequency (∼3 Hz) (Sabatier & Leng 2008, 2010).

Importantly, very few SCN neurons that were non‐responsive to light displayed this harmonic pattern, and so we can conclude that, *in vivo*, it distinguishes light‐responsive cells in the SCN from spontaneously active cells that are not light responsive. We can also conclude from Aggelopoulos & Meissl (2000) that this patterning is not an artefact of the particular anaesthetic conditions, and from Zhang *et al*. (1995) and Brown *et al*. (2008) that it must originate within the SCN itself rather than passively reflecting an oscillatory input from the retina.

In terms of the numbers of light‐responsive SCN cells studied, the present study is the largest to date. Generally, the observations were consistent with previous studies: in particular, light‐on cells outnumbered light‐off cells in all recording conditions by ∼ 2:1, responses to light were consistent within cells but diverged between cells in diverse temporal profiles of responsiveness (Brown *et al*. 2011) and a subset of light‐responsive cells discharged with isoperiodic bursting patterns. In addition, responses to light were stronger at night than during the day, and this was true of both light‐on cells and light‐off cells.

Other studies have consistently reported that most SCN neurons are not light responsive, and this appears in to be contrast to our present findings: the great majority of cells that we recorded were light responsive. For example, Groos & Mason (1980) reported that just 37% of neurones in the caudal SCN of the rat were light responsive and only 17% in the rostral SCN; Meijer *et al*. (1986) reported that just 32% of 205 SCN cells in the rat were light responsive, and Jiao *et al*. (1999) reported that just 27 of 97 SCN cells were light‐responsive. Furthermore, the spontaneous firing rates that we report in the present study are generally higher than reported previously.

These apparent differences may have simple methodological explanations. First, in the present study, unlike most previous studies *in vivo*, we approached the SCN ventrally and tested cells as we encountered them. Thus, we first encountered cells in the ventral SCN, where most retinal afferents terminate. Because we were interested primarily in light‐responsive neurones, which we found abundantly as soon as electrodes entered the SCN, and because we could never be certain that non‐responsive cells found at deeper locations were still within the SCN, in most experiments, we stayed close to where light‐responsive cells had been encountered. Saeb‐Parsy & Dyball (2003) used the same ventral approach to the SCN as us, although they tested very few neurones with light because their study was focussed on relating spontaneous discharge activity to time of day, and aimed to capture the activity across the whole SCN.

Second, there was an inevitable bias in our experiments in favour of recording spontaneously active neurones. Less bias was present in the study of Saeb‐Parsy & Dyball (2003), who recorded from 469 cells in the SCN region with a mean ± SEM rate of 1.81 ± 0.14 spikes s^−1^. Of these, 126 (26.9%) were silent, and were detected only because they could be antidromically activated by stimulating electrodes placed in other hypothalamic regions. Another 54 cells had very slow firing rates of <0.1 spikes s^−1^. We would not have detected these cells.

Single unit recordings from the SCN of freely moving rats would also not have shown such a strong bias towards spontaneously active cells; these involve implanted, immovable electrodes, whereas we were continually tracking to detect active cells. In conscious rats in conditions of constant darkness, Meijer *et al*. (1998) made long recordings from nine SCN neurones of which four were light responsive: their mean discharge rate at subjective night was 0.7 spikes s^−1^ and all fired faster during the day, although at a mean rate of just 1.4 spikes s^−1^. Despite the small number of cells, their study is important in following the activity of single neurones over a prolonged period (>48 h) and thus provides strong evidence of a generalized increase in discharge rates between subjective day and subjective night. We found that light‐off cells were much more active during at night than during the day; this is not discrepant with the observations of Meijer *et al*. (1998) because their small sample did not include any cells inhibited by light, although our observations of light‐on cells are less easy to reconcile. Our sample of 67 light‐on cells recorded in the dark during the day fired at 9.6 ± 0.6 spikes s^−1^ compared to 11.2 ± 0.7 spikes s^−1^ for 86 cells recorded in the dark at subjective night. This is a difference of 1.7 spikes s^−1^ with 95% confidence limits (−0.16 to 3.5) spikes s^−1^, indicating that there is at most a small increase in firing rate of these cells during the day, and more probably a real reduction. The experimental conditions are different, and our data do not include slow firing or silent cells; nevertheless, it appears that, on average, spontaneously active light‐on cells in the SCN of the anaesthetized rat do not fire faster during the subjective day than during the subjective night.

Jiao *et al*. (1999) also found no significant daily rhythm in baseline firing rates of light‐responsive SCN cells, although their sample was small and indicated a trend to increased activity in the day; the mean firing rates of their samples were also much lower than our own. However, they found that SCN cells that did not respond to light were more active during the subjective day than during the night, indicating that the widely‐observed increase in SCN activity during the day involves cells that are not acutely responsive to light. A possibly important methodological difference between the present study and some previous studies (Jiao *et al*. 1999, Brown *et al*. 2011) is that we did not atropinize the eyes because we aimed to study day‐night variation in activity in more closely physiological conditions; thus, the pupillary reflex was intact in our preparations.

Turning to the origin of the harmonic discharge pattern, as argued above, because such a pattern is seen in the SCN *in vitro*, it is probably intrinsically generated in the SCN rather than a response to an oscillatory forcing input from the retina. Against this, Freeman *et al*. (2008) reported that presumptive retinal ganglion cells recorded from the optic chiasm (in urethane‐anaesthetized rats) display harmonic discharge patterning similar the data described in the present study. However, they approached the optic chiasm with fine tungsten microelectrodes from the dorsal surface of the brain, an approach that would have passed through the SCN. They did not mark their recording sites but concluded that they were recording from fibres because of the small tip size of their electrodes. We consider it possible that the units that they recorded were not retinal ganglion fibres but SCN cells or cells in the ventral hypothalamus adjacent to the optic nerve; although the SCN is the most conspicuous aggregation of retinal recipient neurones, photic responses can be recorded in cells adjacent to the optic tract over much of its extent. Conversely, we must consider whether the present recordings were from retinal tract fibres. First, fine‐tipped electrodes do not necessarily record better from small diameter fibres than from cell bodies: fine‐tipped electrodes, as used in the present study, are excellent for recording from magnocellular neurons in the supraoptic nucleus but not from afferent fibres at that site because all units recorded in the supraoptic nucleus can be identified anatomically as projecting to the neural lobe (Leng & Sabatier, 2014). Second, we passed through the optic tract in all experiments, and encountered single units only at depths consistent with penetration of the brain, where units were interspersed with other cells that were not light responsive and which showed different firing patterns. Third, i.c.v. injections of carbenoxolone consistently increased the activity of harmonic cells, an observation that would not be expected if spikes were initiated in the retina. Nevertheless, although we may be confident that the cells described here are SCN cells, we cannot exclude the possibility that their patterning reflects in part a forcing input from the retina, although the abovementioned observations *in vitro* show that this cannot be a complete explanation.

As observed in previous studies, many light‐receptive neurones in the SCN (and some that are light insensitive) display intermittent bursting activity and, in many cases, this bursting appears to be isoperiodic. Interestingly, slow isoperiodic bursting is also seen in light‐responsive neurones in the intergeniculate leaflet (Blasiak & Lewandowski, 2013) and olivary pretectal nucleus (Szkudlarek *et al*. 2008). In other peptidergic neurones, bursting activity optimizes neuropeptide secretion because of non‐linearities in stimulus‐secretion mechanisms at the secretory terminals (Bicknell, 1988). We noted two types of isoperiodic bursting mainly during the subjective day. In light‐on cells, as described by Aggelopoulos & Meissl (2000), isoperiodic bursts usually ended in a fractionated pattern, whereas, in light‐off cells, we observed a different type, in which isoperiodic bursts began with a fractionated pattern. The apparently complementary nature of these patterns, and the similarity in period length for these two patterns, suggests that they may arise from slow mutual inhibition between light‐on cells and light‐off cells.

If the harmonic pattern arises intrinsically in the SCN, how might it be generated? A subpopulation of neurones in the ventromedial nucleus of the hypothalamus also display a harmonic distribution of ISIs but with a much lower fundamental frequency, of ∼3 Hz (Sabatier & Leng, 2010). The ventromedial nucleus is almost exclusively glutamatergic, and these harmonic cells appear to be linked by mutual fast excitation, with the period set by a prolonged activity‐dependent afterhyperpolarization. However, most neurones in the suprachiasmatic nucleus are GABAergic, and so it is more probable that synchronized oscillations arise from mutual inhibitory coupling.

Indeed, synchronized high frequency oscillations (30 Hz and more) occur in many sensory and cortical brain regions, and fast inhibition has been proposed as a general mechanism for their generation. In networks of neurons receiving tonic excitatory input, mutual coupling by inhibitory synapses can lead to synchronous high frequency oscillations that arise from subthreshold oscillations of membrane potential; these enhance synchrony by introducing a depolarization that is mediated by a post‐inhibitory rebound, and this is correlated among neurons due to common inhibitory input. In network models, this can give rise to harmonic ISI distributions similar to those reported in the present study (Baroni *et al*., 2014). Because the primary retinal recipient neurons in the SCN are all GABAergic and subject to an input that is predominantly excitatory, if they are mutually interconnected, then conditions appear to be ripe for synchronization of high frequency oscillatory activity. Second, there appears to be widespread if weak electrotonic coupling amongst SCN neurons (Long *et al*. 2005; Rash *et al*. 2007) that will support synchronization. However, the lack of effect of carbenoxolone on the harmonic discharge patterning suggests that gap junctions may not be essential for harmonic discharge patterning in the SCN. Carbenoxolone is a modestly potent water‐soluble blocker of gap junctions and, at concentrations of 50–100 μm, it mostly eliminates the electrical coupling that occurs among various central neurones. However, it has many other pharmacological actions (Connors, 2012). In particular, it is an antagonist of GABA_A_ receptors (Beaumont & Maccaferri, 2011). Accordingly the increase in discharge rate observed after carbenoxolone may be unrelated to any actions on gap junctions.

Whether the activity of light‐responsive cells in the SCN is globally synchronized, giving rise to a coherent 30 Hz output for which the amplitude is modulated by light intensity, remains to be determined; it is possible that there is only a localized synchronization with multiple subpopulations discharging out of phase with each other. Because both light‐on cells and light‐off cells discharge in rhythms locked to a 30 Hz cycle, an interesting possibility is that the interactions between these populations mean that their discharge is out of phase. If so, then cells receiving a convergent input from light‐on cells and light‐off cells would see a dramatic change in rhythm amplitude in response to light, especially at night.

## Additional information

### Competing interests

The authors declare that they have no competing interests.

### Author contributions

All authors agree to be accountable for all aspects of the work, ensuring that questions related to the accuracy or integrity of any part are appropriately investigated and resolved. All persons designated as authors qualify for authorship, and all those who qualify for authorship are listed. The study was conceived and initially designed by GL and ML. The electrophysiological recordings were made by TT and CT, with support and advice from GL and ML, and data were analysed by TT and GL with assistance from TL. All authors contributed to the writing of the manuscript.

### Funding

Supported by grants from the Biotechnology and Biological Research Council (BB/J004723/1), Medical Research Council (MR/M022838/1) (ML, GL), fellowships from the Japanese Society for the Promotion of Science (TT, CT) and the Scholarship Fund to Study Abroad, SHISEIKAI (CT).

## Supporting information


**Data S1**. Documentation of the data from the present study.
